# Vasculitic neuropathy associated with IgG4-related kidney disease: A case report and literature review 

**DOI:** 10.5414/CN110547

**Published:** 2021-06-04

**Authors:** Benjamin Jiang, Zarife Sahenk, Anjali Satoskar, Miriam Freimer, Isabelle Ayoub

**Affiliations:** 1 The Ohio State University, Department of Neurology,; 2 Nationwide Children’s Hospital, Department of Neurology, Center for Gene Therapy,; 3 The Ohio State University, Department of Pathology, and; 4 The Ohio State University, Department of Medicine, Division of Nephrology, Columbus, OH, USA

**Keywords:** IgG4-related disease, peripheral neuropathy, ANCA-associated vasculitis

## Abstract

IgG4-related disease is an immune-mediated systemic inflammatory condition characterized by tissue infiltration of IgG4-positive plasma cells and elevated serum IgG4 concentrations. Peripheral neuropathy is an atypical manifestation of this disease. We describe an unusual case of vasculitic neuropathy in a patient with IgG4-related kidney disease. A 55-year-old woman presented with right leg weakness progressing to bilateral leg weakness, pain and numbness of the legs, and impaired gait. She was previously evaluated for weight loss and anemia with a CT scan of the abdomen due to concern for malignancy. Abnormal enhancement of the kidneys was seen, and laboratory work-up and kidney biopsy were consistent with IgG4-related disease. Myeloperoxidase-antineutrophil cytoplasmic antibodies were also positive. In combination with the patient’s asymmetric leg weakness and painful neuropathy, this raised concern for vasculitis. Sural nerve biopsy confirmed vasculitic neuropathy. Recent studies have demonstrated an overlap in the clinical characteristics of IgG4-related disease and the anti-neutrophil cytoplasmic antibody-associated vasculitides, which are known to cause vasculitic neuropathy. Clinicians should recognize this association, and IgG4-related disease should be considered in the differential diagnosis in patients with peripheral neuropathy in the right clinical context.

## Introduction 

IgG4-related disease (IgG4-RD) is an immune-mediated systemic inflammatory condition characterized by fibrosis and IgG4-positive plasma cell infiltration of affected tissues and is often associated with elevated serum IgG4 concentrations. First described in patients with autoimmune pancreatitis, it has since been shown to affect multiple organs including kidneys, aorta, lacrimal glands, and salivary glands [1]. Neurologic involvement is less common and typically manifests as pachymeningitis or hypophysitis. Peripheral neuropathy has been reported though it is a rare manifestation [2]. We describe a patient with an initial diagnosis of IgG4-related kidney disease (IgG4-RKD) who presented with painful peripheral neuropathy and was found to have vasculitic neuropathy on sural nerve biopsy. 

## Case description 

**Figure 1.**
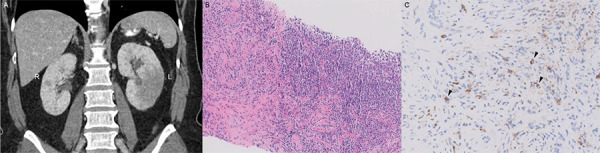
CT scan of the abdomen and kidney biopsy. A: CT scan of the abdomen with iodinated contrast showing asymmetric enlargement of the left kidney, with heterogeneous contrast enhancement and loss of normal corticomedullary differentiation bilaterally. Similar findings were present in the right kidney but to a lesser degree. B: Kidney biopsy showing focally accentuated lymphoplasmacytic inflammatory infiltrates with storiform fibrosis on hematoxylin and eosin stain at × 10 magnification, with (C) IgG4-positive plasma cells (arrowheads) highlighted on immunohistochemical staining for IgG4 on formalin-fixed paraffin-embedded tissue section at × 20 magnification.

A 55-year-old woman with a history of allergic rhinitis and asthma presented with right leg weakness, which then progressed to bilateral leg weakness. She reported 14 kg of unintentional weight loss over the past year, and laboratory evaluation revealed iron deficiency anemia. This was concerning for malignancy and prompted CT scan of the abdomen that demonstrated bilateral enlargement of the kidneys with heterogeneous contrast enhancement and loss of normal corticomedullary differentiation. Kidney biopsy showed storiform fibrosis and plasma cell-rich interstitial inflammation with IgG4 immunostaining showing clusters of IgG4-positive plasma cells (Figure 1), suggestive of IgG4-RKD. Serum creatinine was 1.1 mg/dL. There was no protein or active sediment in the urine. Additional laboratory evaluation (summarized in Table 1) was notable for serum IgG4 level of 177 mg/dL (2.4 – 121 mg/dL), IgE level of 1,309.7 IU/mL (1.5 – 165.3 IU/mL), erythrocyte sedimentation rate (ESR) of 77 (< 30 mm/h), positive myeloperoxidase-antineutrophil cytoplasmic antibodies (MPO-ANCA) by ELISA, and antinuclear antibody ≥ 1 : 1,280. Anti-double stranded DNA (anti-dsDNA) antibody was negative, and complement levels were normal. 

The patient was subsequently seen in neurology clinic prior to initiation of immunosuppressive therapy. By this time, her leg weakness had been present for 8 months, and she also reported 6 months of burning pain and numbness of her lower extremities. Examination was notable for bilateral weakness of ankle dorsiflexion and plantarflexion, loss of pinprick and vibration sense distal to the ankles, and a tentative gait. Electrodiagnostic testing showed a length-dependent sensorimotor axonal polyneuropathy. Given the initially asymmetric pattern of leg weakness, the severity of her pain, and positive MPO-ANCA, there was concern for vasculitic neuropathy. 

**Figure 2.**
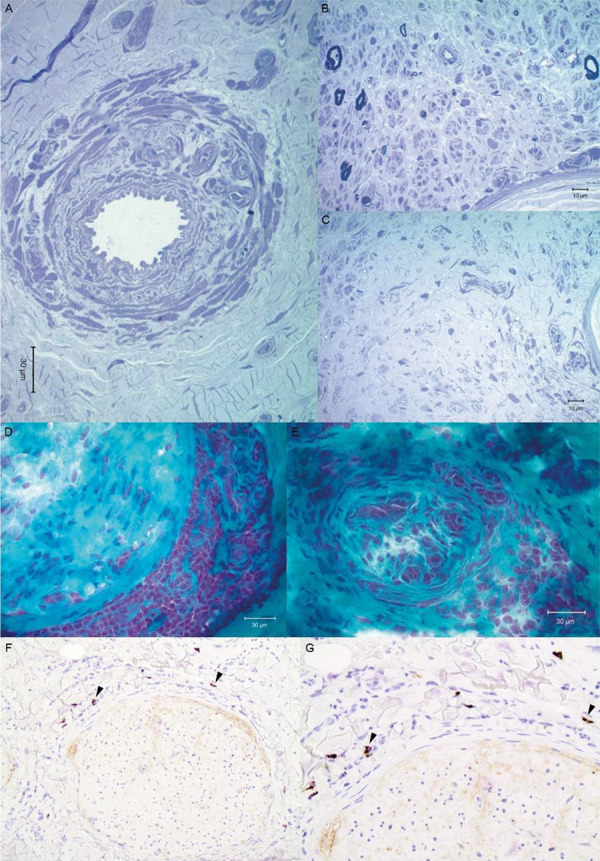
Sural nerve biopsy. A: 1-micron plastic-embedded sections showing a re-canalized vessel in the epineurium and two fascicles showing (B) severe fiber loss and (C) acellularity/fibrosis in the endoneurium. D: Fresh frozen nerve showing inflammation in the perineurium of 1 fascicle and (E) an occluded recanalized vessel with some perivascular mononuclear cells. F: Immunohistochemical staining for IgG4 showing IgG4-positive cells (arrowheads) at × 20 magnification, with (G) × 40 magnification of the same focus.

Biopsy of the left sural nerve was performed, which showed severe myelinated and unmyelinated fiber loss in all fascicles, a recanalized epineurial blood vessel, and dense perineurial mononuclear cell infiltrates, consistent with vasculitic neuropathy. IgG4 immunohistochemical staining demonstrated a few scattered IgG4-positive plasma cells (Figure 2). Baseline positron emission tomography (PET) scan for evaluation of further organ involvement was not approved by the patient’s insurance company. 

The patient was treated with prednisone 40 mg daily and had resolution of neuropathic pain and improvement in gait within 4 weeks. Prednisone was tapered off over a 6-month period. She also received IV immunoglobulin 0.5 g/kg/day for 4 doses then 0.4 g/kg weekly for 8 weeks, followed by rituximab 1 g every 6 months. Subsequent examination demonstrated improvement in lower extremity strength and normal gait. At 6 months of therapy, serum IgG4 level normalized to 57.1 mg/dL, IgE level decreased to 283 IU/mL, and ESR normalized to 25 mm/h. Her kidney function remained normal. 

## Discussion 

The patient’s combination of histopathologic findings, radiographic findings, and elevated serum IgG4 level meet criteria for definite IgG4-RD based on proposed comprehensive diagnostic criteria as well as organ-specific diagnostic criteria for IgG4-related kidney disease [3, 4]. Vasculitic neuropathy is an atypical feature of IgG4-RD. Aortitis can be seen, but inflammation of smaller arteries was previously thought to be rare [1]. However, there is a growing body of literature demonstrating the co-occurrence of IgG4-RD and vasculitis, with or without neuropathy. 

ANCA-associated vasculitides (AAV), which include microscopic polyangiitis (MPA), eosinophilic granulomatosis with polyangiitis (EGPA), and granulomatosis with polyangiitis (GPA), are systemic vasculitides commonly associated with neuropathy that can have clinical characteristics similar to IgG4-RD, and should be considered in the differential diagnosis of IgG4-RD [5]. Recent studies suggest that the similarities extend beyond shared clinical characteristics and have demonstrated ANCA positivity in patients with IgG4-RD, histopathologic and serologic features of IgG4-RD in patients with AAV, and patients diagnosed with both IgG4-RD and AAV concomitantly [5, 6, 7, 8]. 

In one study, researchers examined sural nerve biopsy specimens of 149 patients with neuropathy who had clusters of inflammatory cells on histopathology. 29 patients had IgG4-positive cell infiltration on histopathology, of which 20 also had elevated serum IgG4 levels. 23 of the 29 patients were previously diagnosed with a primary vasculitic neuropathy such as MPA or EGPA [5]. 

Another case report described a patient who was diagnosed with EGPA in the setting of multiple mononeuropathies and purpura, with skin biopsy showing leukocytoclastic vasculitis. The patient also had enlarged lacrimal and submandibular glands and was concomitantly diagnosed with Mikulicz’s disease, a manifestation of IgG4-RD [6]. Other studies have described patients meeting criteria for both IgG4-RD and AAV in the absence of peripheral nerve involvement [7, 8]. 

## Conclusion 

The relationship between IgG4-RD and vasculitic neuropathy has not yet been fully delineated. The patient in this case met criteria for definite IgG4-RD, and also had a vasculitic neuropathy with positive MPO-ANCA. Clinicians should recognize the possibility of this co-occurrence and understand that positive ANCA does not exclude a diagnosis of IgG4-RD, and likewise, an elevated serum IgG4 level does not exclude a diagnosis of AAV. In addition, IgG4-RD should be considered in the differential diagnosis in patients with peripheral neuropathy and evidence of systemic inflammatory disease. [Table Table2]

## Funding 

None. 

## Conflict of interest 

All authors have nothing to declare. 

**Table 1.** Pertinent laboratory results.

Laboratory result	Value	Reference range
Serum creatinine	1.1	0.5 – 1.2 mg/dL
IgG4 level	177	2.4 – 121 mg/dL
IgE level	1309.7	1.5 – 165.3 IU/mL
ESR	77	< 30 mm/h
ANA	≥ 1 : 1,280	None
MPO-ANCA by ELISA	Positive	Negative
Anti-dsDNA antibody	Negative	Negative
C3	151	87 – 200 mg/dL
C4	53	18 – 52 mg/dL

**Table 2. Table2:** Teaching points.

IgG4-RD is an immune-mediated systemic inflammatory condition that can affect the kidneys, nervous system, and many other organs.
IgG4-RD is histopathologically characterized by storiform fibrosis and IgG4-positive plasma cell infiltration of affected tissues, and is often associated with elevated serum IgG4 concentrations.
IgG4-RD can be treated with glucocorticoids as well as other immunomodulatory or immunosuppressive agents.
There is overlap in the presentations of IgG4-RD and ANCA-associated vasculitis.
Consider IgG4-RD in the differential diagnosis in patients with peripheral neuropathy and evidence of systemic inflammatory disease.
